# Stauffer's Syndrome in Patient with Metastatic Prostate Cancer

**DOI:** 10.1155/2019/9745301

**Published:** 2019-04-22

**Authors:** Andris Romašovs, Aldis Puķītis, Viktorija Mokricka, Elina Frolova

**Affiliations:** ^1^University of Latvia, Riga, Latvia; ^2^Center of Gastroenterology, Pauls Stradins Clinical University Hospital, Pilsoņu iela 13, Riga LV-1002, Latvia; ^3^Medical Faculty, Riga Stradins University, Riga, Latvia

## Abstract

Cholestasis is a symptom that can be present in many conditions, such as bile duct obstruction by malignant mases, obstruction by gallstone, acute and chronic viral hepatitis, and autoimmune disorders, such as primary biliary and sclerosing cholangitis. Stauffer syndrome is a rare type of paraneoplastic syndrome that presents as cholestasis with absence of underlying disease. Classically Stauffer syndrome has been described in renal cell cancer patients. In literature cholestasis as manifestation of paraneoplastic syndrome has also been described in patients with prostate adenocarcinoma and pancreatic and bronchogenic carcinoma.* Conclusions*. Stauffer syndrome should be kept in mind in patients who present with cholestasis with no underlying cause. We advise to exclude any possible causes of cholestasis, for example, obstruction of bile duct.

## 1. Introduction

Stauffer syndrome is clinical diagnosis with unknown pathophysiology and incidence rate. Unfortunately there are no known diagnostic criteria. This syndrome was first described by the American gastroenterologist Maurice H. Stauffer, who worked at the Mayo Clinic in Rochester, MN, USA [1]. It was described as paraneoplastic syndrome in a patient with renal cell carcinoma, presenting with cholestatic disease in the absence of underlying liver disease. In a classical case, a patient with Stauffer syndrome would have elevated alkaline phosphatase, GGT (gamma-glutamyl transferase), erythrocyte sedimentation rate, *α*–2 globulin, thrombocytosis, and prolonged prothrombin time [2].

This case study was conducted in accordance with the Declaration of Helsinki. The protocol was approved by the Ethics committee of Pauls Stradins Clinical University Hospital on 8th November, 2018 (No. 081118-15L).

## 2. Case Presentation

An 80-year-old, white Caucasian man was presented to our hospital emergency department with one-month history of jaundice, pruritus, acholic feces, dark urine, unintentional weight loss, and worsening pain in the lumbar region. On the date of admission the urine was light yellow and the patient reported that feces were of normal color and consistence. He denied alcohol or illegal drug use and had no travel history within the last 10 years. He reported jaundice and pruritus progressing for one month prior to admission. He denied having pain, nausea, vomiting, or elevated temperature during the whole month. Pain in the lumbar region was progressing during the last 2 years, due to PCa metastasis. Patient had metastatic PCa and underwent palliative hormonal therapy in ambulatory settings; he also had moderate aortic stenosis found by echocardiography and angina pectoris of class II according to the classification system by Canadian Cardiovascular Society. In year 2016, metastatic process involving the lumbar spinal and thoracic region was diagnosed using skeletal scintigraphy. Starting from year 2016 he received androgen deprivation therapy in outpatient settings and testosterone level was controlled in conjugation with PSA. Testosterone remained at castrate level.

During physical examination, yellow skin, scratch marks on the back and thorax due to pruritus, and aortic murmur were revealed.

Blood tests indicated that the patient had cholestasis, elevated alanine aminotransferase, elevated aspartate transaminase, elevated gamma-glutamyl transferase, and elevated bilirubin (Table 1). Infectious disease such as hepatitis C and hepatitis B was excluded using testing for antibodies. Autoimmune causes were also excluded; anti-mitochondrial antibody, anti-glycoprotein antibody-210, and anti-sp100 antibodies, IgA and IgM, were all negative. Alpha-fetoprotein was also negative. Alkaline phosphatase (ALP) was significantly elevated; thus isoenzyme of bone origin ALP2 was determined to differentiate the source of elevated ALP (Table 1). Ultrasound examination of abdomen was performed, with no evidence of extrahepatic bile duct dilatation or cirrhosis. Magnetic resonance cholangiopancreatography showed no cholestasis, but 1.5 cm diameter cyst was found in hepatic parenchyma. The patient received supportive and symptomatic therapy.

Rapid deterioration of general health status occurred during hospitalization, with exitus letalis while in the hospital.

## 3. Discussion

Our data showed that the patient had the rare and underreported Stauffer syndrome. We have performed extensive investigation to determine a possible reversible cause of cholestasis in this case.

Most commonly Stauffer syndrome has been described relating to RCC (renal cell carcinoma), but its variants can be found in many different oncological conditions as paraneoplastic manifestation of the disease, for example, pancreatic cancer and prostate cancer (PCa) [3, 4]. It is considered that interleukin 6 has a role in the pathogenesis of paraneoplastic syndrome leading to cholestasis and liver damage with no other known causes [5]. Stauffer syndrome in patients with PCa has been reported in about 100 clinical cases since 1961 [3]. PCa remains one of the most common cancers in men, as one in five men with cancer will have PCa [6].

Recently Munveer et al. have reported a patient with newly diagnosed metastatic prostate cancer who developed paraneoplastic syndrome with cholestasis, and it resolved when androgen deprivation therapy was started. In this case PSA level was also significantly elevated and reached 4,130 ng/ml [7]. Min Kyu Kang et al. report a metastatic prostate cancer patient with paraneoplastic syndrome with cholestatic jaundice and effectiveness of androgen deprivation therapy [8]. In our case, watchful waiting with androgen deprivation therapy was started in outpatient setting and level of testosterone was maintained on castration level. Unfortunately we were unable to perform liver biopsy because of coagulopathy that developed during hospitalization.

The range of differential diagnoses for a patient presenting with cholestasis is very broad, and paraneoplastic syndrome should be kept in mind when the patient has a solid tumor with no metastases in liver or other possible causes of mechanical obstruction. By definition, paraneoplastic syndrome is triggered by the altered immune system response due to the primary tumor [9]. First of all, primary causes of liver damage should be excluded. We believe that patient had rare and underreported Stauffer syndrome, as possible mechanical, autoimmune, and infectious causes of cholestasis have been excluded. Unfortunately in this case we were unable to prolong patient life expectancy due to many comorbidities and acute progression.

## Figures and Tables

**Table 1:** Laboratory findings during hospitalization.

Analysis	Day 1	Day 7	Day 14	Reference range	5 months prior to admission date
ALT*∗*,U/L	235	59	33	10-49	19
AST*∗*, U/L	161	51	88	0-33	32
Bilirubin, umol/L	165	162	299	5-21	13
Direct-bilirubin, umol/L	132	133	237	0-5	4
Indirect-bilirubin, umol/L	33	29	63	0-16	9
Testosterone, ng/dL			12.76	86.49-788.22	
Alkaline phosphatase U/L	2513	1416	934	46-116	1356
Alkaline phosphatase bone fraction (ALP2), U/L			366	5.5-22.9	
LDH*∗*, U/L		350	592	120-246	
GGT*∗*, U/L	1174	515	361	0-72	
CRP*∗*, mg/L	24.67	36.2	267.76	0-5	3.6
IL-6*∗*, pg/mL			57	<3.4	
PSA*∗*, ng/mL		66.01	91.78	0-3.99	
APTT*∗*, sec.	36.3	41.8	46.1	26-36	39.5
Prothrombin index,%	87.6	18.4	20.7	70-120	74.5
INR*∗*	1.06	3.16	2.88	0.8-1.2	1.14
Fibrinogen, g/L	5.73	8.58	8.68	0-0.5	1.68

*∗*ALT- alanine aminotransferase; AST- aspartate transaminase; GGT- gamma-glutamyl transferase; CRP- C reactive protein; IL-6- interleukin 6; LDH- lactate dehydrogenase; PSA – prostate specific antigen; APTT – activated partial thromboplastin time; INR – international normalized ratio of prothrombin time of blood coagulation.
